# Vitamin D in Myalgic Encephalomyelitis/Chronic Fatigue Syndrome After COVID-19 or Vaccination: A Randomized Controlled Trial

**DOI:** 10.3390/nu18030521

**Published:** 2026-02-03

**Authors:** Shinichiro Kodama, Mitsuko Nakata, Nafuko Konishi, Masato Yoshino, Akinori Fujisawa, Mutsuo Naganuma, Yuki Kobayashi, Yuriko Hirai, Akiko Kitagawa, Mariko Miyokawa, Ryo Mishima, Satoshi Teramukai, Masanori Fukushima

**Affiliations:** 1 Kodama Clinic, Hyogo 6650842, Japan; 2 Departments of Biostatistics, Graduate School of Medical Science, Kyoto Prefectural University of Medicine, Kyoto 6028566, Japan; 3 Viola Clinic, Osaka 5300044, Japan; 4 Kamata Yoshino Clinic, Tokyo 1440052, Japan; 5 Honbetsu Cardiovascular Medicine Clinic, Hokkaido 0893314, Japan; 6 Tokachi Mutsumino Clinic, Hokkaido 0800020, Japan; 7 Kobayashi Clinic, Hyogo 6580048, Japan; 8 MCL Corporation, Kyoto 6008191, Japan; 9 Kitaris Co., Ltd., Aichi 4820018, Japan; 10 The Clinical and Translational Research Center, Kyoto Prefectural University of Medicine, Kyoto 6028566, Japan; 11 Foundation of Learning Health Society Institute, Nagoya 4500003, Japan

**Keywords:** COVID-19, post-vaccination syndrome, PASC, Long COVID, myalgic encephalomyelitis/chronic fatigue syndrome, vitamin D, COVID-19 vaccine

## Abstract

**Background**: Myalgic encephalomyelitis/chronic fatigue syndrome (ME/CFS) can develop as post-vaccination syndrome (PVS) or Post-Acute Sequelae of SARS-CoV-2 infection (PASC). In our prior retrospective study, most patients with PVS who developed ME/CFS had vitamin D insufficiency or deficiency. We evaluated the efficacy of vitamin D replacement therapy guidance for ME/CFS symptom improvement in patients with vitamin D insufficiency or deficiency. **Methods**: This open-label randomized controlled trial enrolled 91 participants with ME/CFS as PVS or PASC and serum 25(OH) vitamin D < 30 ng/mL across five clinical sites. Participants were randomized 1:1 to intervention (active vitamin D preparation plus vitamin D replacement therapy guidance: 25 μg daily supplementation, dietary counseling, sun exposure, and exercise) or control (active vitamin D preparation alone) for 12 weeks. The primary endpoint was the change in ME/CFS symptom count from screening to Week 12. **Results**: Mean symptom change was −6.7 in the intervention group versus −1.2 in the control group (between-group difference −5.6; 95% CI: −7.2, −3.9; *p* < 0.001). Serum 25(OH) vitamin D improved from 18.6 to 27.1 ng/mL in the intervention group, while the control group showed a decreasing trend (between-group difference 10.2 ng/mL; 95% CI: 7.9, 12.5). Achievement of <8 symptoms (i.e., no longer meeting ME/CFS diagnostic criteria) was significantly higher in the intervention group, with 16 participants achieving this threshold compared to 1 in the control group (*p* < 0.001). Subgroup analyses showed consistent benefit in both PVS (*n* = 56) and PASC (*n* = 29) cohorts. **Conclusions**: Vitamin D replacement therapy guidance significantly reduced ME/CFS symptoms along with improvement of serum 25(OH) vitamin D levels in patients with vitamin D insufficiency or deficiency who developed ME/CFS as PVS or PASC.

## 1. Introduction

Coronavirus disease 2019 (COVID-19) has affected people worldwide since it was declared a pandemic by the World Health Organization (WHO) on 11 March 2020 [1]. As the COVID-19 pandemic has prolonged, so-called Long COVID, or Post-Acute Sequelae of SARS-CoV-2 infection (PASC), has been increasingly reported, characterized by symptoms that usually occur 3 months from the onset of infection and last for at least 2 months [2].

Symptoms of PASC include post-exertional malaise, fatigue, brain fog, dizziness, gastrointestinal symptoms, palpitations, changes in sexual desire or function, loss or alteration of smell or taste, thirst, chronic cough, chest pain, and abnormal movements [3]. The constellation of symptoms in PASC shares features similar to myalgic encephalomyelitis/chronic fatigue syndrome (ME/CFS), which is also thought to occur frequently after viral infections [4].

A safe and effective prophylactic vaccine was urgently needed to contain the COVID-19 pandemic, which had devastating effects on healthcare, the economy, and society [5]. COVID-19 vaccines were subsequently granted Emergency Use Authorization (EUA) by the US Food and Drug Administration (FDA) and have been recognized as effective in reducing severe disease and mortality. However, as vaccination programs expanded rapidly worldwide, numerous adverse events following vaccination have been reported [6]. While direct causal relationships have not been established in many cases, more and more individuals have developed persistent symptoms following vaccination that warrant further investigation.

In a prior study, the authors provided vitamin D replacement therapy guidance for patients with ME/CFS following COVID-19 vaccination who had insufficient or deficient serum 25(OH) vitamin D levels. In that study, 28 patients diagnosed with ME/CFS after COVID-19 vaccination who had deficient or insufficient serum 25(OH) vitamin D levels were instructed to increase sun exposure and consume vitamin D-rich foods (including supplements). As a result, 23 of 28 patients (82.1%) no longer met the diagnostic criteria for ME/CFS [7].

This prior study was a retrospective case series report, and a prospective randomized controlled clinical trial was needed to establish the efficacy of vitamin D replacement therapy guidance for ME/CFS following COVID-19 vaccination. However, we recognized that the therapeutic benefit might extend beyond post-vaccination cases. Similarities in pathophysiology and clinical manifestations have been noted among ME/CFS, PASC, and post-COVID-19 vaccination syndrome [8], suggesting a shared underlying mechanism. Supporting this hypothesis, patients with Long COVID (i.e., PASC) have been reported to have lower serum 25(OH) vitamin D levels compared to COVID-19 patients without Long COVID [9], and similarly, reduced serum 25(OH) vitamin D levels have been reported in ME/CFS [10]. Therefore, we hypothesized that symptomatic improvement through vitamin D replacement therapy guidance could be expected in both post-vaccination and PASC cases, warranting their combined investigation in a single trial. Based on these considerations, we conducted an open-label randomized controlled trial to confirm the efficacy of vitamin D replacement therapy guidance on improvement of ME/CFS symptoms in patients who developed ME/CFS as PASC or following COVID-19 vaccination and had insufficient or deficient serum 25(OH) vitamin D levels. The trial compared active vitamin D preparation plus vitamin D replacement therapy guidance versus active vitamin D preparation alone.

## 2. Methods

### 2.1. Study Design

This study is a randomized, open-label, multicenter trial. The study evaluated the efficacy of adding vitamin D replacement therapy guidance to alfacalcidol, the only active vitamin D preparation approved by the Ministry of Health, Labour and Welfare (MHLW) in Japan, for improving symptoms in patients with serum 25(OH) vitamin D levels < 30 ng/mL who developed ME/CFS as PVS or PASC.

Patient enrollment began on 2 December 2024, and ended on 30 March 2025, with each patient receiving treatment for 24 weeks after their enrollment. This report presents results at the 12-week primary efficacy evaluation time point.

Eligible participants were patients aged 18 years or older at the time of consent who had vitamin D insufficiency or deficiency (serum 25(OH) vitamin D levels < 30 ng/mL) and met the diagnostic criteria for ME/CFS (2003 Canadian Clinical Case Definition) [11] following COVID-19 infection or vaccination. Symptom assessment was conducted using a checklist based on these diagnostic criteria [11], following standardized procedures to ensure consistency of evaluation across study sites.

**Figure 1:**
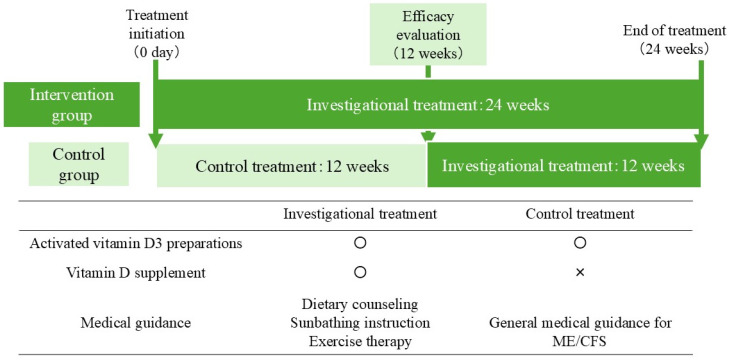
Schema of investigational and control treatments. Details of the investigational and control treatments and the treatment schema for each group are provided. The intervention group will receive 24 weeks of investigational treatment, and the control group will receive 12 weeks of control treatment followed by investigational treatment. ○, included in the treatment; ×, not included in the treatment.

The investigational treatment and control treatment schemas are shown in Figure 1. The investigational treatment was defined as a combination of active vitamin D preparation and vitamin D replacement therapy guidance. The active vitamin D preparation was alfacalcidol (Onealpha^®^ tablets, 1.0 μg, once daily) [Teijin Pharma Limited, Tokyo, Japan] [12], and vitamin D replacement therapy guidance consisted of vitamin D supplementation, dietary guidance, sun exposure guidance, and exercise therapy as directed by the attending physician. The vitamin D supplement was NATUREMADE Super Vitamin D (25 μg, once daily) [Otsuka Pharmaceutical Co., Ltd., Tokyo, Japan]. The control treatment was defined as a combination of active vitamin D preparation alone and general guidance. The active vitamin D preparation was alfacalcidol (Onealpha^®^ tablets, 1.0 μg, once daily), and general guidance for ME/CFS was provided. The intervention group received investigational treatment for 24 weeks. The control group received the control treatment for 12 weeks, followed by the investigational treatment for 12 weeks to observe the response after switching.

The primary endpoint was the change in the number of ME/CFS symptoms from screening to Week 12. The secondary endpoints were: achievement of <8 ME/CFS symptoms (i.e., no longer meeting ME/CFS diagnostic criteria) at Week 12, change in the number of ME/CFS symptoms from screening to Week 24, change in serum 25(OH) vitamin D levels from screening to Week 12, change in serum 25(OH) vitamin D levels from screening to Week 24, change in performance status from screening to Week 12, and change in serum albumin levels from screening to Week 12.

This report presents the primary endpoint and the secondary endpoints evaluable at 12 weeks.

Vitamin D insufficiency was defined as serum 25(OH) vitamin D levels < 30 ng/mL and ≥20 ng/mL at screening, and vitamin D deficiency was defined as serum 25(OH) vitamin D levels < 20 ng/mL at screening [13]. All 25(OH)D measurements at the primary study site were performed by FALCO biosystems Ltd. [Kyoto, Japan], using the ECLIA method (Elecsys Vitamin D total II, Roche Diagnostics [Tokyo, Japan]), which is approved by the Japanese Ministry of Health, Labour and Welfare as the standard testing method in Japanese clinical settings. The remaining sites used accredited clinical laboratories with approved methods for 25(OH)D measurement. Cases with a history of COVID-19 vaccination before the onset of ME/CFS were classified as PVS (post-vaccination syndrome), and cases with a history of COVID-19 infection before the onset of ME/CFS were classified as PASC (Post-Acute Sequelae of COVID-19). For cases with both vaccination and infection before ME/CFS onset, classification was based on the event closest to ME/CFS onset.

### 2.2. Sample Size Calculation

Based on results from our previous study [7], the mean change in the number of ME/CFS symptoms at 90 days without vitamin D replacement therapy guidance (*n* = 5) was estimated to be −3.0, serving as the assumed mean change for the control group. With vitamin D replacement therapy guidance (*n* = 23), the mean change was estimated to be −4.6, serving as the assumed mean change for the intervention group. The common standard deviation for the mean change was estimated to be 2.5 based on data from patients who received vitamin D replacement therapy guidance in the previous study. Using a two-sample t-test with a two-sided significance level of 0.05, a sample size of at least 80 patients (40 per group) would provide a power of 0.8. The target enrollment was set at 90 patients (45 per group), anticipating some exclusions from analysis.

### 2.3. Randomization

Study participants were randomized 1:1 to the intervention group and control group using stratified block randomization. Stratification factors were study site and number of ME/CFS symptoms at screening (≤10, ≥11).

For randomization, the investigator submitted a registration form to the case registration center. After confirming there were no discrepancies in the content, the case registration center performed treatment allocation using the randomization list and communicated the result to the investigator via the registration form on which the allocation result was recorded. The randomization list was maintained centrally at the case registration center.

### 2.4. Safety Monitoring

Throughout the study period, serum calcium, phosphate, and creatinine levels were measured at baseline and at regular follow-up visits (Weeks 4, 8, 12, and 24). These measurements were performed using standard clinical laboratory methods at each participating site. Renal function was assessed using serum creatinine and estimated glomerular filtration rate (eGFR).

If serum calcium exceeded the upper limit of the institutional reference range, alfacalcidol dose reduction or temporary discontinuation would be implemented at the discretion of the investigator, with prompt reassessment of laboratory values.

### 2.5. Data Collection and Management

All study-related data for study participants were recorded by the investigator in paper-based case report forms (CRFs) based on source documents. CRF data were managed and reviewed at the data center according to the data management plan. For inconsistent data, queries were issued to the study sites, and corrections were made based on documented responses.

Quality control of the collected data was performed through off-site monitoring and central monitoring.

### 2.6. Statistical Analysis

Baseline characteristics were summarized for each treatment group. For the primary endpoint, which is the change in the number of ME/CFS symptoms from screening to Week 12, estimation was performed using mixed models for repeated measures (MMRM) with the change in the number of ME/CFS symptoms as the response variable; participants as the random effect; and treatment group, time point (Weeks 4, 8, and 12), the interaction between treatment and time point, and the number of ME/CFS symptoms at screening as fixed effects. An unstructured covariance matrix was used to model within-subject variance. The intervention group was considered superior to the control group if the upper limit of the two-sided 95% confidence interval (CI) for the between-group difference in mean change from screening in the number of ME/CFS symptoms at Week 12 was less than zero. As a sensitivity analysis, missing data at Week 12 were imputed using the last observation carried forward (LOCF) method. The secondary endpoints are analyzed as follows; the change from screening in serum 25(OH) vitamin D levels at week 12 was analyzed similarly as the primary endpoint; achievement of <8 ME/CFS symptoms at Week 12 was compared between groups using the Cochran–Mantel–Haenszel test stratified by the number of ME/CFS symptoms at screening (≤10, ≥11). For the primary endpoint, subgroup analyses were performed by the number of ME/CFS symptoms at screening (≤10, ≥11) and by PVS/PASC classification.

We conducted post hoc analysis as follows: we performed a time-to-event analysis using time to have fewer than eight symptoms as response variable and baseline characteristics and laboratory data as explanatory variables. Univariable screening (selection threshold: *p* < 0.2) and multivariable Cox regression analysis (backward stepwise selection method, selection threshold: *p* < 0.1) were used to identify potential risk factors; Symptom improvement rates were compared between treatment groups by Fisher’s exact test.

All statistical tests were two-sided, and a *p*-value < 0.05 was considered statistically significant. All *p*-values, except for the primary analysis of the primary endpoint, are exploratory in nature. All analyses used SAS version 9.4 (SAS Institute, Inc., Cary, NC, USA).

## 3. Results

**Figure 2:**
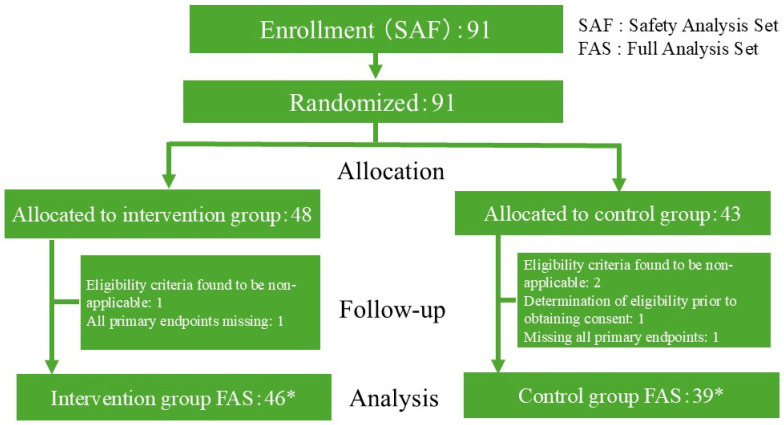
Flowchart of study participants. A flow chart of the study participants is provided. Of the target sample size of 90 patients, 91 patients were randomized and 3 patients were found not to meet the eligibility criteria, 1 patient was judged eligibility before consent was obtained, and 2 patients were missing the primary endpoint. As a result, a total of 85 patients, 46 in the intervention group and 39 in the control group, were included in the full analysis set. * Of the 85 participants in the FAS, 80 completed the Week 12 assessment (42 intervention, 38 control). Four participants discontinued (3 intervention, 1 control), and 1 participant in the intervention group had missing Week 12 data.

From 2 December 2024 to 30 March 2025, 91 participants were enrolled from 5 sites (Figure 2). Of the 91 enrolled participants (Safety Analysis Set [SAF]), 48 were assigned to the intervention group and 43 to the control group. Three participants were found not to meet eligibility criteria after allocation, 1 participant had eligibility assessed before obtaining consent, and 2 participants had missing data for the primary endpoint at all time points (Weeks 4, 8, and 12). Excluding these participants, a total of 85 participants (46 in the intervention group and 39 in the control group) formed the Full Analysis Set (FAS).

### 3.1. Participant Characteristics

Baseline characteristics of the 85 participants in the FAS are shown in Table 1. The cohort comprised 30 men (35.3%) and 55 women (64.7%) with a mean age of 49.5 (SD 19.7) years and a median time from symptom onset of 983 days (range: 3–1490 days). Fifty-six participants (65.9%) were classified as PVS and 29 (34.1%) as PASC. A history of COVID-19 infection was present in 58 participants (68.2%), and a history of COVID-19 vaccination in 75 (88.2%).

### 3.2. Temporal Changes in Laboratory Values and Symptoms

**Figure 3:**
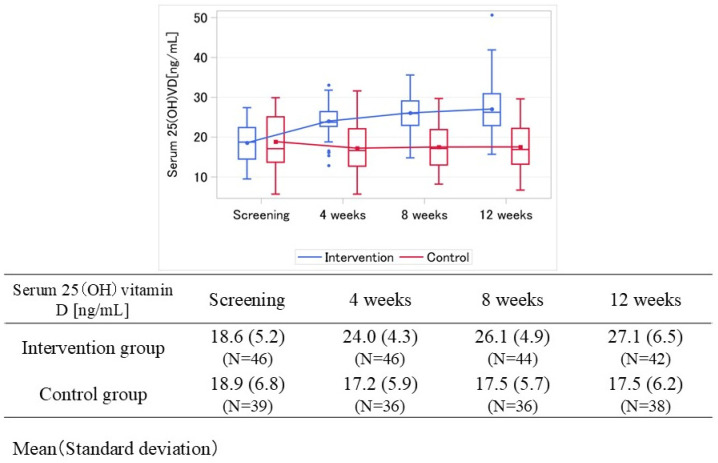
Longitudinal trend in serum 25(OH) vitamin D by treatment groups. Box-and-whisker plots of serum 25 (OH) vitamin D concentration (ng/mL) over time for each treatment group is shown, with blue representing the intervention group and red the control group.

**Figure 4:**
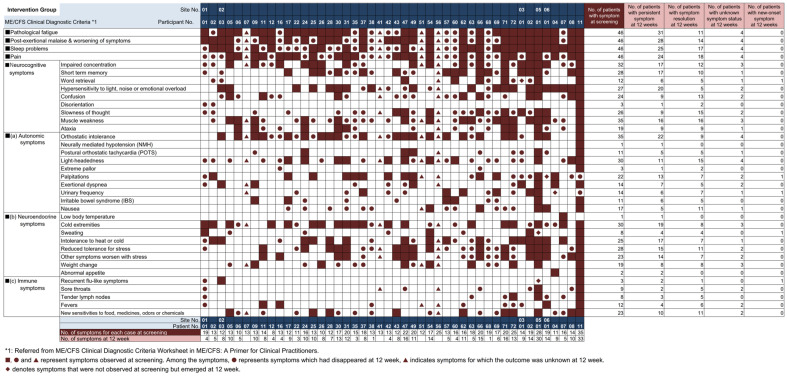
ME/CFS symptoms at screening and 12 weeks (Intervention group). Symptoms observed in the intervention groups at screening and at 12 weeks are listed by participant. ■ ● ▲ indicates symptoms present at screening, ■ indicates continued at 12 weeks, ● indicates resolved at 12 weeks, ▲ indicates unknown, and ◆ indicates symptoms not present at screening but occurring at 12 weeks.

**Figure 5:**
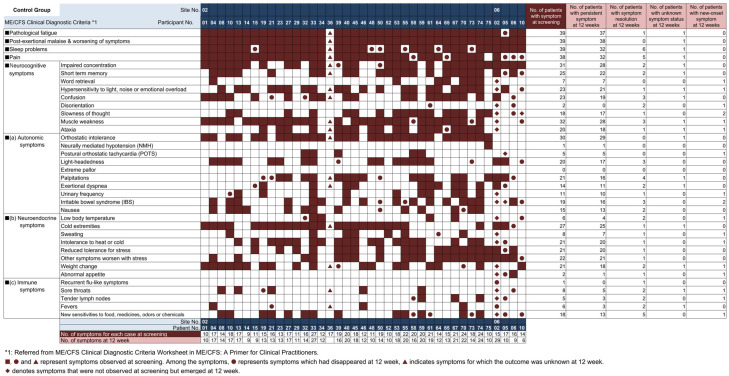
ME/CFS symptoms at screening and 12 weeks (Control group). Symptoms observed in the control groups at screening and at 12 weeks are listed by participant. ■ ● ▲ indicates symptoms present at screening, ■ indicates continued at 12 weeks, ● indicates resolved at 12 weeks, ▲ indicates unknown, and ◆ indicates symptoms not present at screening but occurring at 12 weeks.

Figure 3 shows the time course of serum 25(OH) vitamin D levels for both groups. Mean serum 25(OH) vitamin D levels at screening were 18.6 (SD 5.2) ng/mL in the intervention group and 18.9 (SD 6.8) ng/mL in the control group. At Week 12, levels improved to 27.1 (SD 6.5) ng/mL in the intervention group, while levels in the control group showed no improvement, remaining at 17.5 (SD 6.2) ng/mL. Figure 4 and Figure 5 show changes in ME/CFS symptoms from screening to Week 12 in the intervention group and control group, respectively. At screening, all participants had pathologic fatigue, sleep problems, and autonomic symptoms. The intervention group had a total of 739 symptoms present at screening, of which 286 resolved at 12 weeks, with 61.3% of symptoms persisting (including unknown status). The control group had a total of 638 symptoms present at screening, of which 64 resolved at 12 weeks, with 90.0% of symptoms persisting (including unknown status). At Week 12, the intervention group had a lower proportion of persistent symptoms compared to the control group.

### 3.3. Primary Endpoint Results

**Figure 6:**
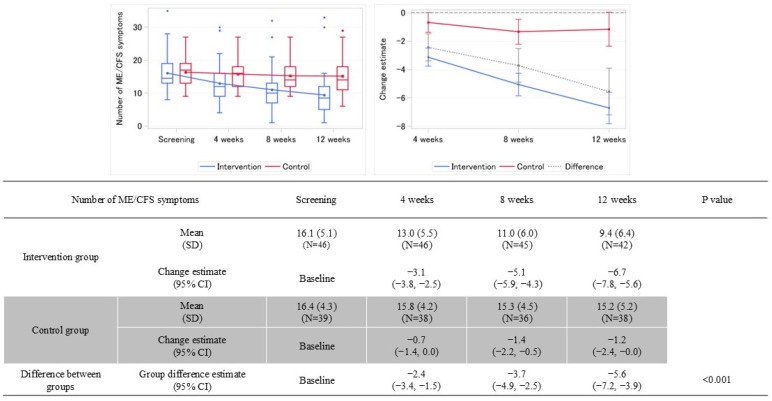
Number of ME/CFS symptoms by treatment groups (Left: longitudinal trend, Right: MMRM estimates of change from screening). Left figure: box-and-whisker plots of the number of ME/CFS symptoms over time. Right figure: MMRM estimated change from screening (solid line: mean; error bars: 95% confidence interval) for each treatment group: blue for treatment, red for control, and gray for difference between groups. In the table, the dark shaded rows indicate the control group.

Figure 6 shows the primary endpoint, the change from screening in the number of ME/CFS symptoms at Week 12. The mean number of ME/CFS symptoms at screening was 16.1 (SD 5.1) in the intervention group and 16.4 (SD 4.3) in the control group. At Week 12, the mean number of symptoms was 9.4 (SD 6.4) in the intervention group and 15.2 (SD 5.2) in the control group. The MMRM-estimated mean change from screening in the number of ME/CFS symptoms at Week 12 was −6.7 (95% CI: −7.8, −5.6) in the intervention group and −1.2 (95% CI: −2.4, 0.0) in the control group, with a between-group difference of −5.6 (95% CI: −7.2, −3.9; *p* < 0.001). The LOCF sensitivity analysis yielded similar results.

### 3.4. Secondary Endpoints Results

Table 2 shows the proportion of participants achieving <8 ME/CFS symptoms (i.e., no longer meeting ME/CFS diagnostic criteria) at Week 12. Among participants with ≤10 symptoms at screening, 2 of 3 (66.7%) in the intervention group and 0 of 4 (0%) in the control group achieved <8 symptoms. Among participants with ≥11 symptoms at screening, 14 of 39 (35.9%) in the intervention group and 1 of 34 (2.9%) in the control group achieved <8 symptoms. The proportion achieving <8 symptoms was higher in the intervention group in both strata, with a statistically significant difference by stratified analysis (*p* < 0.001).

**Figure 7:**
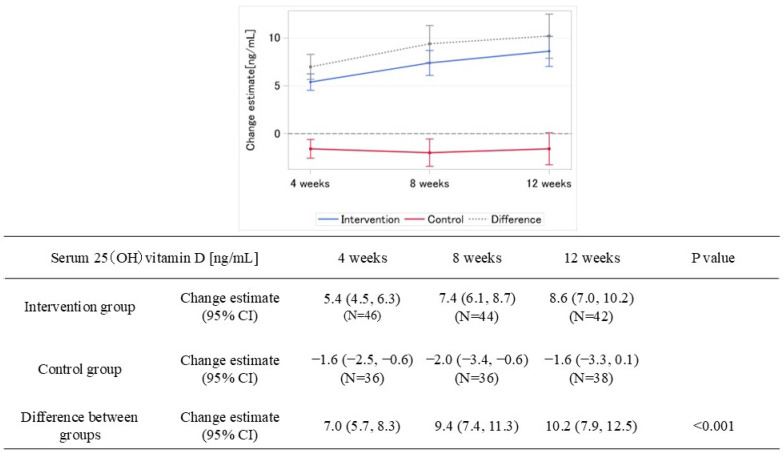
MMRM estimates of change from baseline in serum 25(OH) vitamin D by treatment groups. MMRM estimates of change in serum 25(OH)vitamin D concentration [ng/mL] from screening (solid line: mean; error bars: 95% confidence interval) for each treatment group: blue for treatment, red for control, and gray for difference between groups.

Figure 7 shows the change from screening in serum 25(OH) vitamin D levels at Week 12. The MMRM-estimated mean change from screening in serum 25(OH) vitamin D levels at Week 12 was 8.6 ng/mL (95% CI: 7.0, 10.2) in the intervention group and −1.6 ng/mL (95% CI: −3.3, 0.1) in the control group, with a between-group difference of 10.2 ng/mL (95% CI: 7.9, 12.5).

### 3.5. Subgroup Analyses

Supplementary Figures S1–S4 show the results of subgroup analyses. In the subgroup with ≤10 ME/CFS symptoms at screening (*n* = 7), the intervention group demonstrated greater reduction in the number of ME/CFS symptoms compared with the control group (between-group difference −3.5 [95% CI: −5.1, −1.9]). A similar effect was observed in the subgroup with ≥11 symptoms at screening (*n* = 78; between-group difference −5.6 [95% CI: −7.4, −3.9]). The intervention was effective in both the PVS subgroup (*n* = 56; between-group difference −6.4 [95% CI: −8.2, −4.6]) and the PASC subgroup (*n* = 29; between-group difference −3.7 [95% CI: −7.1, −0.2]).

### 3.6. Safety Evaluation

Supplementary Table S1 lists all adverse events. Serious adverse events were assessed in the SAF (*n* = 91). One participant in the control group experienced two serious adverse events: acute pancreatitis and pancreatic cancer. A causal relationship with the active vitamin D preparation was ruled out. The clinical outcome is unknown.

No cases of severe hypercalcemia or clinically significant renal function impairment were observed during the study period, and all laboratory abnormalities were reversible with appropriate management.

### 3.7. Symptoms at Screening and Week 12

Table 3 shows the number of participants and symptom improvement rates by ME/CFS diagnostic criteria, with detailed symptom-level breakdowns provided in Supplementary Table S2. In the intervention group, immune symptoms showed the highest improvement rate (45.5%), followed by pain symptoms (39.1%) and sleep problems (37.0%). For most symptom categories, the intervention group showed higher improvement rates compared with the control group at Week 12.

### 3.8. Factors Affecting Time to Achievement of <8 ME/CFS Symptoms

Table 4 shows the results of multivariable Cox regression analysis for time to achievement of <8 ME/CFS symptoms. Stepwise selection identified baseline symptom count, treatment allocation, PVS/PASC classification, comorbidities, mean corpuscular hemoglobin concentration (MCHC), and total bilirubin (T-Bil) as factors associated with time to <8 symptoms. The intervention group achieved <8 symptoms significantly faster than the control group (hazard ratio for control vs. intervention, 0.06; 95% CI: 0.01, 0.43). Additionally, higher MCHC was associated with a lower likelihood of achieving <8 symptoms (hazard ratio per 1% increase, 0.51; 95% CI: 0.30, 0.85).

## 4. Discussion

In this study, we evaluated whether correcting vitamin D insufficiency or deficiency would improve ME/CFS symptoms in patients who developed ME/CFS as PVS or PASC. We compared active vitamin D preparation combined with vitamin D replacement therapy guidance versus active vitamin D preparation alone over 12 weeks.

ME/CFS is a complex chronic disease characterized by a spectrum of symptoms including pathologic fatigue, post-exertional malaise, cognitive dysfunction, immune dysfunction, unrefreshing sleep, pain, autonomic dysfunction, and neuroendocrine symptoms [14]. Although the etiology and pathogenesis of ME/CFS have not yet been clearly defined, research indicates that viral infections are the most common trigger; other contributing factors include genetic predisposition, sex, vaccination, physical trauma, and immune system dysfunction or hypoactivity [15]. In fact, approximately half of patients with PASC are estimated to meet the criteria for ME/CFS [16].

Although the cause of PASC remains speculative, recent reports suggest that SARS-CoV-2 spike protein may be associated with immune dysfunction and neuroinflammation [17]; similarly, spike proteins produced following COVID-19 vaccination may affect the immune system. The symptoms of ME/CFS developing after COVID-19 vaccination and SARS-CoV-2 infection may involve common immunologic pathways and may represent symptoms of “Spikeopathy” [18]. Recent research strongly suggests that autoantibodies may be involved in the neurological symptoms of PASC [19,20], with spike protein potentially serving as a trigger for aberrant autoimmune responses. Notably, autoantibody-mediated mechanisms have also been documented in ME/CFS more broadly; a study revealed the relationship between anti-β1/anti-β2 adrenergic receptor antibody titers and intracerebral structural network abnormalities [21], suggesting that autoimmune processes may represent a common pathogenic pathway in ME/CFS regardless of initial trigger. Supporting this autoimmune hypothesis, ME/CFS patients demonstrate a skewed B cell receptor repertoire associated with infection-related disease onset, which may drive pathogenic autoantibody production; this BCR repertoire skewing has been reported to have potential as a biomarker for ME/CFS [22]. We proposed that ME/CFS following COVID-19 vaccination and PASC may develop through similar mechanisms involving spike protein-triggered autoimmune responses. Therefore, as our previous study demonstrated the efficacy of vitamin D replacement therapy guidance for ME/CFS following COVID-19 vaccination, we hypothesized that vitamin D replacement therapy guidance should also be effective for PASC.

Supporting this hypothesis, vitamin D has been shown to have effects on neuropsychiatric symptoms of Long COVID through modulating both immune and neuronal cells [23]. Mechanistically, vitamin D exerts immunomodulatory effects including promotion of regulatory T cell development [24], suppression of B cell differentiation and autoantibody production [25], and inhibition of dendritic cell maturation leading to immune tolerance [26]. The urgency of this study is further underscored by the current situation, including petitions to the Japanese National Diet by patients with PASC seeking intractable disease designation, and the lack of established treatment for ME/CFS despite multiple reports of the condition following COVID-19 vaccination [7,27].

The vitamin D deficiency underlying many ME/CFS cases is itself pandemic-scale. Amid reports of vitamin D being linked to a variety of diseases, including cardiovascular and autoimmune diseases and chronic musculoskeletal pain [28,29], Holick warned in 2010 that more than 1 billion people worldwide had vitamin D insufficiency or deficiency [30]. In fact, a meta-analysis conducted in the US and Europe in 2012 found that 86% of 4383 subjects were deficient in vitamin D [31]. In 2019, it was estimated that approximately 75% of adults worldwide had serum 25(OH) vitamin D levels below 30 ng/mL [32]. In Japan, serum 25(OH) vitamin D was measured in 5518 individuals who underwent medical checkups from 2019 to 2020, and 98% were found to have vitamin D insufficiency or deficiency [33]. Similarly, comparable patterns were identified in 2021 among healthcare professionals [34], suggesting that even those with medical knowledge are not immune to this nutritional challenge.

One reason this widespread deficiency in a fundamental nutrient has persisted largely unrecognized is a startling revelation: a significant statistical error in the Recommended Dietary Allowance (RDA) calculation method published by the Institute of Medicine [35], which has led to vitamin D intake guidelines that substantially underestimate actual requirements. Japan’s guidelines are no exception [36]. The clinical impact of inadequate vitamin D status is also evident in COVID-19 outcomes. Patients who developed Long COVID after COVID-19 infection were reported to have significantly lower serum 25(OH) vitamin D levels [9]; conversely, vitamin D3 supplementation has been reported to potentially reduce the risk of COVID-19-related ICU admission and mortality [37]. Additionally, vitamin D has been shown to have protective effects against COVID-19-induced organ damage across multiple systems, including cardiovascular, neurological, renal, hepatic, and immune organs [38]. These findings indicate that the entire population is vulnerable to preventable diseases, making revision of vitamin D RDA guidelines an urgent priority.

Given this widespread deficiency and its implications for ME/CFS, we sought to determine the optimal approach to vitamin D correction in affected patients. Building on our previous study in which we provided vitamin D replacement therapy guidance to 28 patients who developed ME/CFS following COVID-19 vaccination and had vitamin D insufficiency or deficiency [7], this study examined whether alfacalcidol, the only active vitamin D preparation approved by MHLW in Japan (administered as Onealpha^®^ tablets, 1.0 μg once daily), could effectively treat ME/CFS, or whether vitamin D supplementation at the dose used safely in our previous study could provide treatment benefit.

In this study, at 12 weeks, the intervention group showed a significant decrease in the number of ME/CFS symptoms accompanying the increase in serum 25(OH) vitamin D levels, whereas the control group receiving alfacalcidol (Onealpha^®^ tablets) showed no significant increase in serum 25(OH) vitamin D levels and no significant decrease in ME/CFS symptoms. These findings demonstrate that inactive vitamin D3 supplementation (25 μg) effectively elevated serum 25(OH) vitamin D levels, and that ME/CFS symptoms significantly decreased accompanying the increase in serum 25(OH) vitamin D levels.

Regarding the differential effects on serum 25(OH) vitamin D levels, alfacalcidol (Onealpha^®^ tablets) contains 1.0 μg of active vitamin D3, whereas the vitamin D3 supplement contains 25 μg of inactive vitamin D3. Although absorption and activation processes affect final serum concentrations, the substantial dose difference likely contributes to the observed effect. Inactive vitamin D3 has been shown to be more effective than active vitamin D3 in raising serum 25(OH) vitamin D levels [39]. Furthermore, active vitamin D is physiologically regulated by parathyroid hormone (PTH) and undergoes renal activation control; however, this regulatory mechanism is impaired in patients with renal dysfunction, leading to increased risk of hypercalcemia with active vitamin D supplementation [40]. Therefore, considering both the efficiency of raising serum 25(OH) vitamin D levels and the risk profile for adverse events, we conclude that treatment with inactive vitamin D3 is preferable.

When we analyzed subgroups within the FAS (56 participants in the PVS group and 29 in the PASC group), the PVS group demonstrated a larger between-group treatment difference in ME/CFS symptom reduction, suggesting that vitamin D replacement therapy guidance may be more effective for PVS. However, this finding may be influenced by unequal and limited sample sizes and by our definition of PASC in this study, so it should be interpreted as exploratory and hypothesis-generating rather than definitive. Specifically, we classified participants not based on their complete vaccination or infection history, but rather based on whether their most recent triggering event before ME/CFS onset was COVID-19 vaccination or SARS-CoV-2 infection. Therefore, we cannot completely exclude the possibility of misclassification—participants in the PASC group may have been affected by prior vaccination, and those in the PVS group may have been affected by prior infection—and further investigation is warranted.

The similarity in symptoms between PVS and PASC may be attributed to spike protein-mediated cellular damage, termed “Spikeopathy,” caused by both coronavirus infection and COVID-19 vaccination. Spike proteins have been shown to induce mitochondrial dysfunction, thereby affecting the cellular powerhouses essential for energy production, cellular repair, and physiological resilience [18]. The pathophysiological and clinical similarities among ME/CFS, PASC, and PVS have been recognized [8], and the relationship between ME/CFS and Long COVID with mitochondrial dysfunction has attracted considerable attention; prolonged symptoms following specific infections may increase the energy demands on the immune system, but mitochondrial dysfunction caused by spike protein impairs the ability to meet these increased demands [41]. Collectively, these findings suggest that both PVS and PASC may involve cellular damage from persistently produced spike protein, leading to mitochondrial dysfunction and diverse clinical manifestations. Spike protein-mediated damage has also been linked to thrombosis [42], and in cases where PASC progresses to ME/CFS, reduced tissue perfusion due to microthrombi and impaired mitochondrial function appear to be involved [43].

In this study, higher baseline MCHC was associated with less symptom improvement. Elevated MCHC has been reported to correlate with reduced red blood cell (RBC) deformability [44], and patients with ME/CFS have been shown to have reduced RBC deformability, which in turn leads to impaired microvascular perfusion and tissue oxygenation, potentially causing diverse symptoms [45]. Participants with elevated MCHC at baseline were less likely to achieve <8 ME/CFS symptoms (i.e., no longer meeting ME/CFS diagnostic criteria), which may be related to reduced RBC deformability. Reduced RBC deformability has been attributed to the effects of reactive oxygen species (ROS) [46]. As discussed above, ME/CFS, PVS, and PASC involve mitochondrial dysfunction; notably, mitochondrial dysfunction and ROS have a strong bidirectional relationship, with mitochondrial dysfunction increasing ROS production, which in turn exacerbates mitochondrial dysfunction [47]. Thus, in PVS and PASC, spike protein-mediated cellular damage and mitochondrial dysfunction may collectively lead to the diverse clinical manifestations as seen in ME/CFS. However, this remains speculative based on the present study, and detailed investigations will be needed to confirm these mechanisms.

Although this study focused on vitamin D among nutrients, we recognize that insufficiency or deficiency of common nutrients in individuals represents a blind spot deeply related to disease onset or progression. For example, COVID-19 severity has been significantly associated with serum zinc concentrations [48]. This nutritional paradigm extends from infectious diseases to intractable neurodegenerative disorders, where individualized nutritional interventions are showing promise. In familial multiple system atrophy (MSA), reduced CoQ10 levels have been observed, and high-dose ubiquinol has shown efficacy [49]; in amyotrophic lateral sclerosis (ALS), ultra-high-dose methylcobalamin has been demonstrated to slow functional decline in patients with early-stage disease and those with moderate progression rates [50], and higher plasma levels of alpha-linolenic acid (ALA), an omega-3 polyunsaturated fatty acid, are associated with longer survival and slower functional decline [51]. In addition to specific nutrient deficiencies, disruption of NAD+ homeostasis has emerged as a common metabolic vulnerability linking diverse neurodegenerative and neuroimmune conditions. The severity of Alzheimer’s disease correlates with dysregulation of NAD+ homeostasis [52], and ME/CFS patients similarly show disruption of the NAD+ de novo pathway with accumulation of 3-hydroxykynurenine [53], suggesting that restoration of NAD+ metabolism may represent a fundamental therapeutic target across multiple neurological disorders.

Collectively, the relationship between nutritional status and disease is critically important. Given that nutritional status can affect disease severity as demonstrated above, awareness of nutritional status should be an integral part of preventive healthcare. If early nutritional interventions can prevent disease progression or mitigate severity, future medical practice should adopt more proactive nutritional strategies. A Japanese study that stratified serum 25(OH) vitamin D levels by season and sex reported an inverse correlation between vitamin D levels and mortality [54]. Similarly, a German analysis demonstrated that promoting vitamin D supplementation among older adults is a cost-saving measure that could significantly reduce cancer mortality, preventing an estimated 30,000 deaths annually [55]. These findings underscore the need to revise current nutritional guidelines to recognize that optimal nutrient levels vary according to individual homeostatic factors (age, sex, geographic location), genetic factors, and individual diseases. Given that pharmacological interventions can carry substantial risk of adverse effects in some cases, we should consider transitioning beyond reliance solely on symptom-focused pharmacological treatments toward treatment approaches that emphasize biological foundations and harness the body’s inherent capacity for health maintenance and healing.

## 5. Limitations of the Study

Because this study employed an open-label design, the effects of placebo response, observer bias, expectancy effects, and reporting bias cannot be completely excluded, and these factors may have influenced the results, potentially inflating perceived benefits. However, the nature of the intervention—which included behavioral components such as dietary guidance, sun exposure recommendations, and exercise therapy—presented inherent challenges to blinding. Complete blinding would have been difficult to implement in this real-world clinical setting.

Furthermore, with 83.5% of participants enrolled from a single site (Kodama Hospital/Kodama Clinic), the effects of enrollment bias and site-specific observer bias cannot be completely excluded. To address this concern, we examined the 14 participants enrolled from sites other than Kodama Hospital/Kodama Clinic separately. Of these 14 participants, 10 were in the intervention group and 4 were in the control group. Although no statistically significant difference was observed in this subgroup (*n* = 14), a trend toward improvement was noted (between-group difference −3.3 [95% CI: −12.4, 5.7]).

Another limitation of this study is the use of a simple symptom count as the primary endpoint, which does not directly reflect symptom severity or quality of life (QOL). However, ME/CFS is characterized by multisystem symptoms affecting multiple organ systems, and the number of concurrent symptoms has clinical relevance in real-world practice. In routine clinical settings, symptom count is widely used for longitudinal monitoring and assessment of treatment response. For an exploratory, real-world-oriented study such as ours, symptom count represents a pragmatic outcome measure that minimizes patient burden while providing clinically interpretable information.

To address these limitations, this study employed a crossover design, in which the control group received the control treatment for 12 weeks, then switched to the investigational treatment (the same comprehensive guidance provided to the intervention group) for the subsequent 12 weeks (Figure 1). If the control group also demonstrates symptom improvement during weeks 12–24 when receiving the comprehensive guidance, this crossover design will provide further evidence supporting the treatment effect beyond the primary 12-week comparison. Additionally, to complement the symptom count endpoint, we evaluated Performance Status change at Week 24 as a measure of functional impact and quality of life. Results through Week 24, including the control group’s crossover response and Performance Status outcomes, will be reported in a separate publication.

Future studies with larger sample sizes, longer follow-up periods, and double-blind placebo-controlled designs are warranted to further confirm the efficacy of vitamin D replacement therapy guidance and to assess the durability of treatment effects in ME/CFS patients developing as PASC or following COVID-19 vaccination.

## 6. Conclusions

In this randomized controlled trial, patients who developed ME/CFS as PVS or PASC and had vitamin D insufficiency or deficiency demonstrated significant symptom reduction along with improvement of serum 25(OH) vitamin D levels when treated with vitamin D replacement therapy guidance, which included daily vitamin D supplementation (25 μg) combined with dietary guidance, sun exposure recommendations, and exercise therapy, compared with alfacalcidol alone. The intervention group showed a mean reduction of 6.7 symptoms at Week 12, whereas the control group showed minimal change. Additionally, significantly more participants in the intervention group achieved <8 symptoms (no longer meeting ME/CFS diagnostic criteria). These findings suggest that correcting vitamin D insufficiency or deficiency through comprehensive vitamin D replacement therapy guidance—including inactive vitamin D3 supplementation with dietary counseling, sun exposure recommendations, and exercise therapy—may represent an effective therapeutic approach for ME/CFS following PASC or COVID-19 vaccination.

However, given that most participants were enrolled from a single site, replication in larger, multicenter studies with more balanced enrollment across geographically and clinically diverse populations is essential to confirm these findings and establish their broader generalizability.

## Figures and Tables

**Table 1:** Baseline Characteristics of Study Participants (by Treatment Group).

			Intervention Group (*n* = 46)	Control Group (*n* = 39)	Total (*n* = 85)
Site	Kamata Yoshino Clinic		2 (4.3%)	0 (0.0%)	2 (2.4%)
	Kodama Hospital/Kodama Clinic		36 (78.3%)	35 (89.7%)	71 (83.5%)
	Honbetsu Cardiovascular Medicine Clinic		2 (4.3%)	0 (0.0%)	2 (2.4%)
	Kobayashi Clinic		1 (2.2%)	0 (0.0%)	1 (1.2%)
	Tokachi Mutsumino Clinic		5 (10.9%)	4 (10.3%)	9 (10.6%)
Number of ME/CFS Symptoms		≤10	3 (6.5%)	4 (10.3%)	7 (8.2%)
		≥11	43 (93.5%)	35 (89.7%)	78 (91.8%)
Sex		Male	17 (37.0%)	13 (33.3%)	30 (35.3%)
		Female	29 (63.0%)	26 (66.7%)	55 (64.7%)
PVS/PASC		PVS	33 (71.7%)	23 (59.0%)	56 (65.9%)
		PASC	13 (28.3%)	16 (41.0%)	29 (34.1%)
Age at consent (years)		Mean (SD)	48.0 (19.6)	51.3 (19.8)	49.5 (19.7)
Days since onset		Median (range)	1040 (211–1284)	891 (3–1490)	983 (3–1490)
Height (cm)		Mean (SD)	161.9 (8.8)	160.4 (10.5)	161.2 (9.5)
Weight (kg)		Mean (SD)	60.3 (12.5)	57.0 (13.4)	58.8 (12.9)
BMI (kg/m^2^)		Mean (SD)	22.9 (3.7)	22.0 (3.8)	22.5 (3.8)
Comorbidities		No	26 (56.5%)	21 (53.8%)	47 (55.3%)
		Yes	20 (43.5%)	18 (46.2%)	38 (44.7%)
Medical history		No	37 (80.4%)	29 (74.4%)	66 (77.6%)
		Yes	9 (19.6%)	10 (25.6%)	19 (22.4%)
COVID-19 infection history		No	15 (32.6%)	12 (30.8%)	27 (31.8%)
		Yes	31 (67.4%)	27 (69.2%)	58 (68.2%)
Number of infections		1 time	24 (77.4%)	19 (70.4%)	43 (74.1%)
		2 times	7 (22.6%)	7 (25.9%)	14 (24.1%)
		3 times	0 (0.0%)	1 (3.7%)	1 (1.7%)
Time from most recent infection to consent		≤3 months	3 (9.7%)	6 (22.2%)	9 (15.5%)
		4–6 months	4 (12.9%)	2 (7.4%)	6 (10.3%)
		7–12 months	3 (9.7%)	6 (22.2%)	9 (15.5%)
		>1 year	21 (67.7%)	10 (37.0%)	31 (53.4%)
		Unknown	0 (0.0%)	3 (11.1%)	3 (5.2%)
COVID-19 vaccination history		No	4 (8.7%)	6 (15.4%)	10 (11.8%)
		Yes	42 (91.3%)	33 (84.6%)	75 (88.2%)
Number of vaccinations		1 time	5 (11.9%)	0 (0.0%)	5 (6.7%)
		2 times	14 (33.3%)	8 (24.2%)	22 (29.3%)
		3 times	11 (26.2%)	14 (42.4%)	25 (33.3%)
		4 times	12 (28.6%)	11 (33.3%)	23 (30.7%)
Time from most recent vaccination to consent		7–12 months	0 (0.0%)	1 (3.0%)	1 (1.3%)
		>1 year	42 (100.0%)	32 (97.0%)	74 (98.7%)
Vaccine manufacturer		Pfizer (Comirnaty)	34 (81.0%)	29 (87.9%)	63 (84.0%)
		Takeda/Moderna (Moderna/Spikevax)	12 (28.6%)	8 (24.2%)	20 (26.7%)
		Moderna (Spikevax bivalent)	4 (9.5%)	3 (9.1%)	7 (9.3%)
		Other vaccine	1 (2.4%)	0 (0.0%)	1 (1.3%)
		Unknown	1 (2.4%)	5 (15.2%)	6 (8.0%)

**Table 2:** Achievement of <8 ME/CFS Symptoms at Week 12.

Symptoms at Screening	Achievement of <8	Intervention Group (*n* = 42)	Control Group (*n* = 38)	Total (*n* = 80)	*p*-Value *
≤10 (N = 7) †	Yes	2 (66.7%)	0 (0.0%)	2 (28.6%)	<0.001
	No	1 (33.3%)	4 (100.0%)	5 (71.4%)	
≥11 (N = 73) †	Yes	14 (35.9%)	1 (2.9%)	15 (20.5%)	
	No	25 (64.1%)	33 (97.1%)	58 (79.5%)	

* *p*-value from Cochran–Mantel–Haenszel test. † By Week 12, 5 participants had discontinued (4 from the intervention group and 1 from the control group), with 80 participants completing the Week 12 assessment.

**Table 3:** Number of Patients and Symptom Improvement Rates by ME/CFS Diagnostic Criteria.

ME/CFS Clinical Diagnostic Criteria	Patients with Symptoms at Screening		Patients with Continued Symptoms at Week 12		Patients with Improved Symptoms at Week 12		Patients with Unknown Symptoms at Week 12		Symptom Improvement Rate		*p*-Value *
	Intervention Group	Control Group	Intervention Group	Control Group	Intervention Group	Control Group	Intervention Group	Control Group	Intervention Group	Control Group	
Pathological fatigue	46	39	31	37	11	1	4	1	23.9%	2.6%	0.005
Post-exertional malaise & worsening of symptoms	46	39	28	38	14	0	4	1	30.4%	0.0%	<0.001
Sleep problems	46	39	25	32	17	6	4	1	37.0%	15.4%	0.03
Pain	46	38	24	32	18	5	4	1	39.1%	13.2%	0.01
Neurocognitive symptoms	46	39	37	38	5	0	4	1	10.9%	0.0%	0.06
Autonomic symptoms	46	39	33	37	9	1	4	1	19.6%	2.6%	0.02
Neuroendocrine symptoms	46	39	35	38	7	0	4	1	15.2%	0.0%	0.01
Immune symptoms	33	25	14	18	15	6	4	1	45.5%	24.0%	0.11

* *p*-value from Fisher’s exact test.

**Table 4:** Multivariable Cox Regression Analysis for Time to Achievement of <8 ME/CFS Symptoms.

		Hazard Ratio	95% CI	*p*-Value
Number of symptoms at baseline	Per symptom	0.87	0.74–1.01	0.07
Treatment group	Control group vs. Intervention group	0.06	0.01–0.43	0.006
PVS/PASC	PASC vs. PVS	0.29	0.07–1.09	0.07
Comorbidities	Yes vs. No	0.29	0.09–0.89	0.03
MCHC	Per 1%	0.51	0.30–0.85	0.010
T-Bil	Per 1 mg/dL	7.03	0.99–50.0	0.05

## Data Availability

The original contributions presented in this study are included in the article and Supplementary Materials. Further inquiries can be directed to the corresponding author.

## Supplementary Materials

The following supporting information can be downloaded at: https://www.mdpi.com/article/10.3390/nu18030521/s1, Table S1. List of Adverse Events, Table S2. ME/CFS Symptoms by Treatment Group, Figure S1. ME/CFS symptoms by treatment groups (Number of symptoms less than 11 at screening), Figure S2. ME/CFS symptoms by treatment groups (Number of symptoms more than or equal to 11 at screening), Figure S3. ME/CFS symptoms by treatment groups (PVS), Figure S4. ME/CFS symptoms by treatment groups (PASC).
