# Supplementary material for: Vitamin D in Myalgic Encephalomyelitis/Chronic Fatigue Syndrome After COVID-19 or Vaccination: A Randomized Controlled Trial
Source: Nutrients. 2026 Feb 3;18(3):521. doi: 10.3390/nu18030521 (PMC12899809; doi:10.3390/nu18030521)
Supplement: Supplementary file 1 [file nutrients-18-00521-s001.zip › 4_Ver2_Clean_SupplementaryTables_EN.pdf]

Supplementary Table S1. List of Adverse Events

Treatment group	Event name	SOC*	PT*	Seriousness	Basis for seriousness	Relationship to alfacalcidol	Severity	Treatment administered	Outcome
Control group (same patient)	Acute pancreatitis	Gastrointestinal disorders	Pancreatitis acute	Serious	(3) requires inpatient hospitalisation or prolongation of existing hospitalisation	Unrelated	Severe	None	Unknown
	Pancreatic cance	Neoplasms benign, malignant and unspecified (incl cysts and polyps)	Pancreatic carcinoma	Serious	(2) is life-threatening	Unrelated	Severe	None	Unknown

*SOC, System Organ Class; PT, Preferred Term. Adverse events were coded using MedDRA version 28.0.

Supplementary Table S2. ME/CFS Symptoms by Treatment Group

	Screening		Week 4		Week 8		Week 12	
	Intervention Group (N=46)	Control Group (N=39)	Intervention Group (N=46)	Control Group (N=38)	Intervention Group (N=45)	Control Group (N=36)	Intervention Group (N=42)	Control Group (N=38)
Pathological fatigue	46 (100.0 %)	39 (100.0 %)	41 (89.1 %)	38 (100.0 %)	39 (86.7 %)	36 (100.0 %)	31 (73.8 %)	37 (97.4 %)
Post-exertional malaise & worsening of symptoms	46 (100.0 %)	39 (100.0 %)	41 (89.1 %)	38 (100.0 %)	35 (77.8 %)	36 (100.0 %)	28 (66.7 %)	38 (100.0 %)
Sleep problems	46 (100.0 %)	39 (100.0 %)	37 (80.4 %)	34 (89.5 %)	28 (62.2 %)	30 (83.3 %)	25 (59.5 %)	32 (84.2 %)
Pain	46 (100.0 %)	38 (97.4 %)	36 (78.3 %)	35 (92.1 %)	31 (68.9 %)	32 (88.9 %)	24 (57.1 %)	32 (84.2 %)
Neurocognitive symptoms	46 (100.0 %)	39 (100.0 %)	46 (100.0 %)	38 (100.0 %)	45 (100.0 %)	36 (100.0 %)	37 (88.1 %)	38 (100.0 %)
Impaired concentration	32 (69.6 %)	31 (79.5 %)	30 (65.2 %)	30 (78.9 %)	26 (57.8 %)	26 (72.2 %)	17 (40.5 %)	28 (73.7 %)
Short term memory	28 (60.9 %)	25 (64.1 %)	23 (50.0 %)	22 (57.9 %)	19 (42.2 %)	22 (61.1 %)	17 (40.5 %)	22 (57.9 %)
Word retrieval	12 (26.1 %)	7 (17.9 %)	11 (23.9 %)	9 (23.7 %)	9 (20.0 %)	8 (22.2 %)	7 (16.7 %)	8 (21.1 %)
Hypersensitivity to light, noise or emotional overload	27 (58.7 %)	23 (59.0 %)	24 (52.2 %)	22 (57.9 %)	24 (53.3 %)	19 (52.8 %)	20 (47.6 %)	22 (57.9 %)
Confusion	24 (52.2 %)	23 (59.0 %)	13 (28.3 %)	22 (57.9 %)	9 (20.0 %)	19 (52.8 %)	9 (21.4 %)	19 (50.0 %)
Disorientation	3 (6.5 %)	2 (5.1 %)	2 (4.3 %)	2 (5.3 %)	0 (0.0 %)	1 (2.8 %)	1 (2.4 %)	1 (2.6 %)
Slowness of thought	26 (56.5 %)	18 (46.2 %)	20 (43.5 %)	18 (47.4 %)	14 (31.1 %)	20 (55.6 %)	9 (21.4 %)	19 (50.0 %)
Muscle weakness	35 (76.1 %)	32 (82.1 %)	29 (63.0 %)	30 (78.9 %)	20 (44.4 %)	27 (75.0 %)	16 (38.1 %)	29 (76.3 %)
Ataxia	19 (41.3 %)	20 (51.3 %)	15 (32.6 %)	19 (50.0 %)	10 (22.2 %)	18 (50.0 %)	9 (21.4 %)	19 (50.0 %)
Autonomic symptoms	46 (100.0 %)	39 (100.0 %)	42 (91.3 %)	38 (100.0 %)	37 (82.2 %)	36 (100.0 %)	33 (78.6 %)	37 (97.4 %)
Orthostatic intolerance	35 (76.1 %)	30 (76.9 %)	28 (60.9 %)	30 (78.9 %)	26 (57.8 %)	29 (80.6 %)	22 (52.4 %)	29 (76.3 %)

	Screening		Week 4		Week 8		Week 12	
	Intervention Group (N=46)	Control Group (N=39)	Intervention Group (N=46)	Control Group (N=38)	Intervention Group (N=45)	Control Group (N=36)	Intervention Group (N=42)	Control Group (N=38)
Neurally mediated hypotension (NMH)	1 (2.2 %)	1 (2.6 %)	0 (0.0 %)	1 (2.6 %)	1 (2.2 %)	1 (2.8 %)	1 (2.4 %)	1 (2.6 %)
Postural orthostatic tachycardia (POTS)	11 (23.9 %)	5 (12.8 %)	9 (19.6 %)	5 (13.2 %)	8 (17.8 %)	5 (13.9 %)	5 (11.9 %)	6 (15.8 %)
Light-headedness	30 (65.2 %)	20 (51.3 %)	24 (52.2 %)	19 (50.0 %)	18 (40.0 %)	17 (47.2 %)	11 (26.2 %)	17 (44.7 %)
Extreme pallor	3 (6.5 %)	0 (0.0 %)	0 (0.0 %)	0 (0.0 %)	1 (2.2 %)	0 (0.0 %)	1 (2.4 %)	0 (0.0 %)
Palpitations	22 (47.8 %)	21 (53.8 %)	18 (39.1 %)	20 (52.6 %)	17 (37.8 %)	15 (41.7 %)	14 (33.3 %)	16 (42.1 %)
Exertional dyspnea	14 (30.4 %)	14 (35.9 %)	11 (23.9 %)	12 (31.6 %)	7 (15.6 %)	10 (27.8 %)	7 (16.7 %)	11 (28.9 %)
Urinary frequency	14 (30.4 %)	11 (28.2 %)	11 (23.9 %)	11 (28.9 %)	9 (20.0 %)	11 (30.6 %)	7 (16.7 %)	11 (28.9 %)
Irritable bowel syndrome (IBS)	11 (23.9 %)	19 (48.7 %)	10 (21.7 %)	18 (47.4 %)	7 (15.6 %)	15 (41.7 %)	6 (14.3 %)	18 (47.4 %)
Nausea	17 (37.0 %)	15 (38.5 %)	12 (26.1 %)	13 (34.2 %)	6 (13.3 %)	15 (41.7 %)	5 (11.9 %)	13 (34.2 %)
Neuroendocrine symptoms	46 (100.0 %)	39 (100.0 %)	45 (97.8 %)	38 (100.0 %)	38 (84.4 %)	36 (100.0 %)	35 (83.3 %)	38 (100.0 %)
Low body temperature	1 (2.2 %)	6 (15.4 %)	1 (2.2 %)	5 (13.2 %)	1 (2.2 %)	4 (11.1 %)	1 (2.4 %)	5 (13.2 %)
Cold extremities	30 (65.2 %)	27 (69.2 %)	28 (60.9 %)	26 (68.4 %)	23 (51.1 %)	23 (63.9 %)	19 (45.2 %)	25 (65.8 %)
Sweating	8 (17.4 %)	8 (20.5 %)	7 (15.2 %)	7 (18.4 %)	6 (13.3 %)	8 (22.2 %)	5 (11.9 %)	8 (21.1 %)
Intolerance to heat or cold	25 (54.3 %)	21 (53.8 %)	24 (52.2 %)	21 (55.3 %)	21 (46.7 %)	20 (55.6 %)	17 (40.5 %)	21 (55.3 %)
Reduced tolerance for stress	28 (60.9 %)	21 (53.8 %)	23 (50.0 %)	21 (55.3 %)	20 (44.4 %)	20 (55.6 %)	15 (35.7 %)	20 (52.6 %)
Other symptoms worsen with stress	23 (50.0 %)	22 (56.4 %)	20 (43.5 %)	21 (55.3 %)	18 (40.0 %)	20 (55.6 %)	14 (33.3 %)	21 (55.3 %)
Weight change	19 (41.3 %)	21 (53.8 %)	12 (26.1 %)	19 (50.0 %)	9 (20.0 %)	17 (47.2 %)	8 (19.0 %)	19 (50.0 %)
Abnormal appetite	2 (4.3 %)	2 (5.1 %)	1 (2.2 %)	2 (5.3 %)	1 (2.2 %)	1 (2.8 %)	2 (4.8 %)	2 (5.3 %)

	Screening		Week 4		Week 8		Week 12	
	Intervention Group (N=46)	Control Group (N=39)	Intervention Group (N=46)	Control Group (N=38)	Intervention Group (N=45)	Control Group (N=36)	Intervention Group (N=42)	Control Group (N=38)
Immune symptoms	33 (71.7 %)	25 (64.1 %)	26 (56.5 %)	21 (55.3 %)	23 (51.1 %)	18 (50.0 %)	14 (33.3 %)	18 (47.4 %)
Recurrent flu-like symptoms	3 (6.5 %)	1 (2.6 %)	3 (6.5 %)	0 (0.0 %)	2 (4.4 %)	0 (0.0 %)	3 (7.1 %)	0 (0.0 %)
Sore throats	9 (19.6 %)	8 (20.5 %)	4 (8.7 %)	8 (21.1 %)	5 (11.1 %)	4 (11.1 %)	2 (4.8 %)	6 (15.8 %)
Tender lymph nodes	8 (17.4 %)	5 (12.8 %)	3 (6.5 %)	3 (7.9 %)	3 (6.7 %)	3 (8.3 %)	3 (7.1 %)	4 (10.5 %)
Fevers	12 (26.1 %)	6 (15.4 %)	7 (15.2 %)	4 (10.5 %)	7 (15.6 %)	2 (5.6 %)	4 (9.5 %)	3 (7.9 %)
New sensitivities to food, medicines, odors or chemicals	23 (50.0 %)	18 (46.2 %)	18 (39.1 %)	15 (39.5 %)	16 (35.6 %)	15 (41.7 %)	10 (23.8 %)	14 (36.8 %)
