# Supplementary material for: Vitamin D in Myalgic Encephalomyelitis/Chronic Fatigue Syndrome After COVID-19 or Vaccination: A Randomized Controlled Trial
Source: Nutrients. 2026 Feb 3;18(3):521. doi: 10.3390/nu18030521 (PMC12899809; doi:10.3390/nu18030521)
Supplement: Supplementary file 1 [file nutrients-18-00521-s001.zip › 5_SupplementaryFigureCaptions_3.pdf]

Supplementary Figure S1. ME/CFS symptoms by treatment groups (Number of symptoms less than 11 at screening)

(Left: longitudinal trend, Right : MMRM estimates of change from screening)

Left figure : box-and-whisker plots of the number of ME/CFS symptoms over time. Right figure : MMRM estimated change from screening (solid line: mean; error bars: 95% confidence interval) for each treatment group: blue for treatment, red for control, and gray for difference between groups.

Supplementary Figure S2. ME/CFS symptoms by treatment groups (Number of symptoms more than or equal to 11 at screening)

(Left: longitudinal trend, Right : MMRM estimates of change from screening)

Left figure : box-and-whisker plots of the number of ME/CFS symptoms over time. Right figure : MMRM estimated change from screening (solid line: mean; error bars: 95% confidence interval) for each treatment group: blue for treatment, red for control, and gray for difference between groups.

Supplementary Figure S3. ME/CFS symptoms by treatment groups (PVS)

(Left: longitudinal trend, Right : MMRM estimates of change from screening)

Left figure : box-and-whisker plots of the number of ME/CFS symptoms over time. Right figure : MMRM estimated change from screening (solid line: mean; error bars: 95% confidence interval) for each treatment group: blue for treatment, red for control, and gray for difference between groups.

Supplementary Figure S4. ME/CFS symptoms by treatment groups (PASC)

(Left: longitudinal trend, Right : MMRM estimates of change from screening)

Left figure : box-and-whisker plots of the number of ME/CFS symptoms over time. Right figure : MMRM estimated change from screening (solid line: mean; error bars: 95% confidence interval) for each treatment group: blue for treatment, red for control, and gray for difference between groups.
