# Supplementary figures and images for: Vitamin D in Myalgic Encephalomyelitis/Chronic Fatigue Syndrome After COVID-19 or Vaccination: A Randomized Controlled Trial
Source: Nutrients. 2026 Feb 3;18(3):521. doi: 10.3390/nu18030521 (PMC12899809; doi:10.3390/nu18030521)

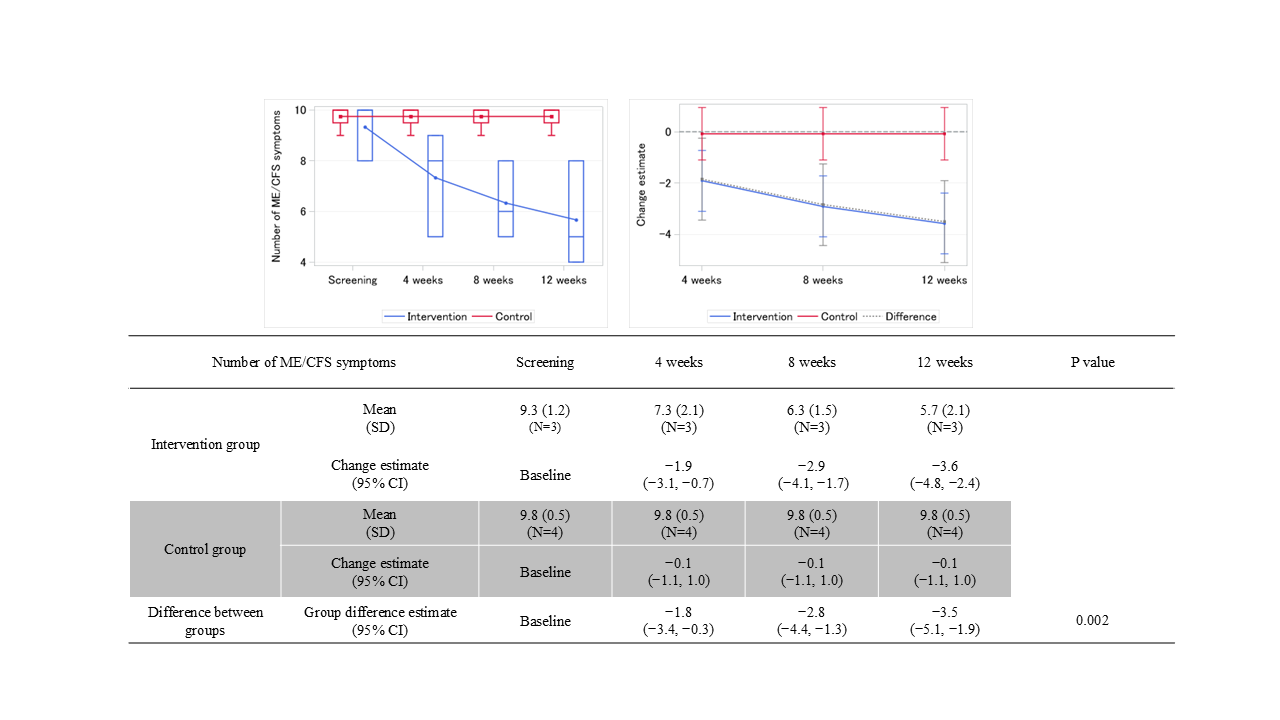

Supplement: Supplementary file 1 [file nutrients-18-00521-s001.zip › SupplementaryFigureS1.PNG]

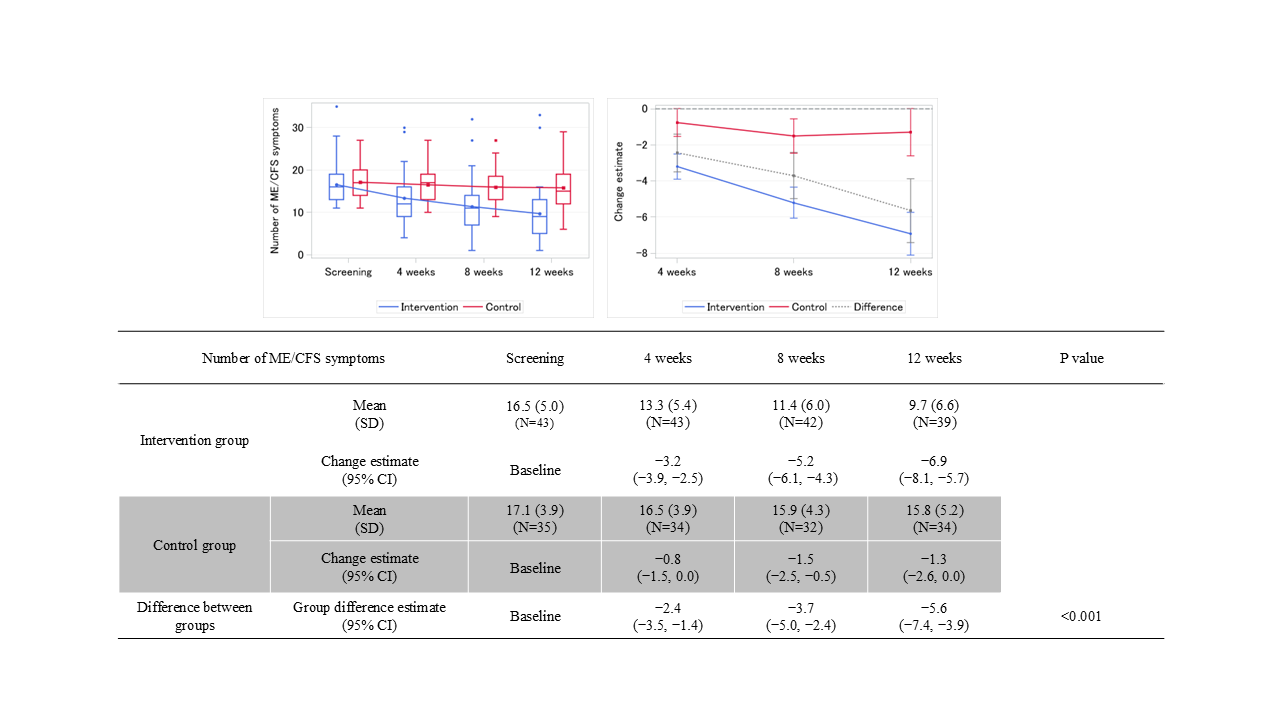

Supplement: Supplementary file 1 [file nutrients-18-00521-s001.zip › SupplementaryFigureS2.PNG]

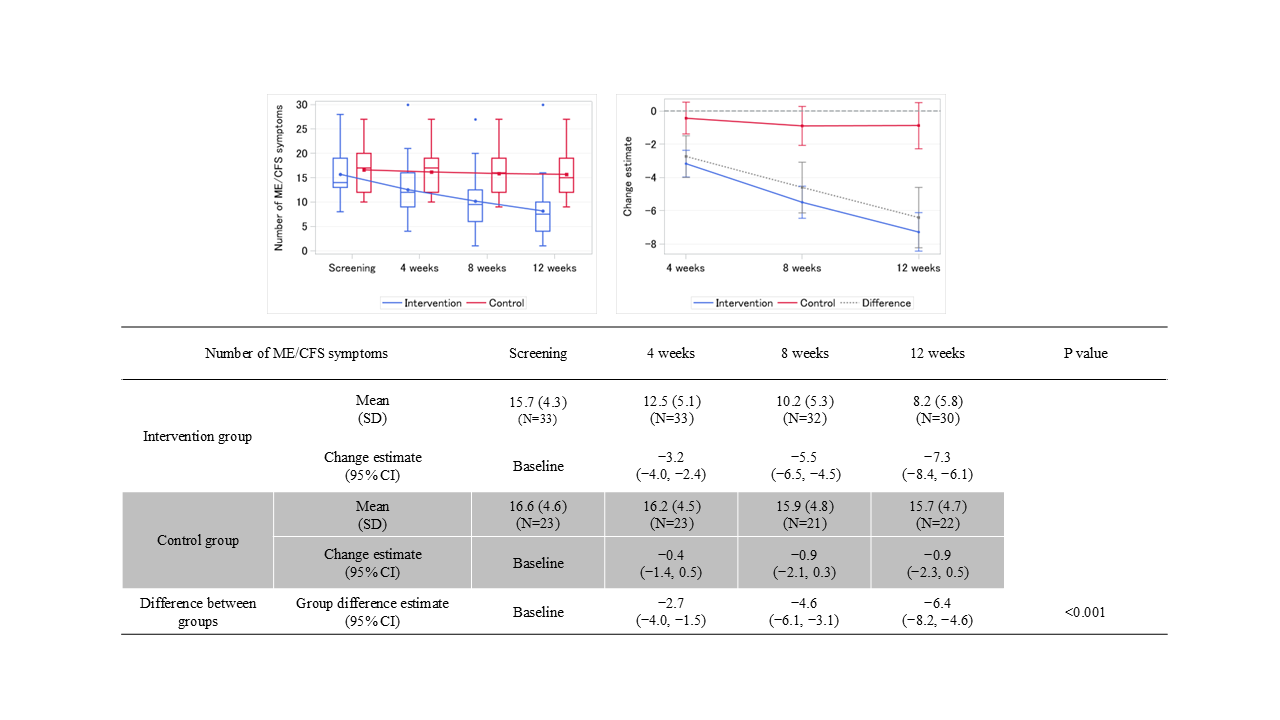

Supplement: Supplementary file 1 [file nutrients-18-00521-s001.zip › SupplementaryFigureS3.PNG]

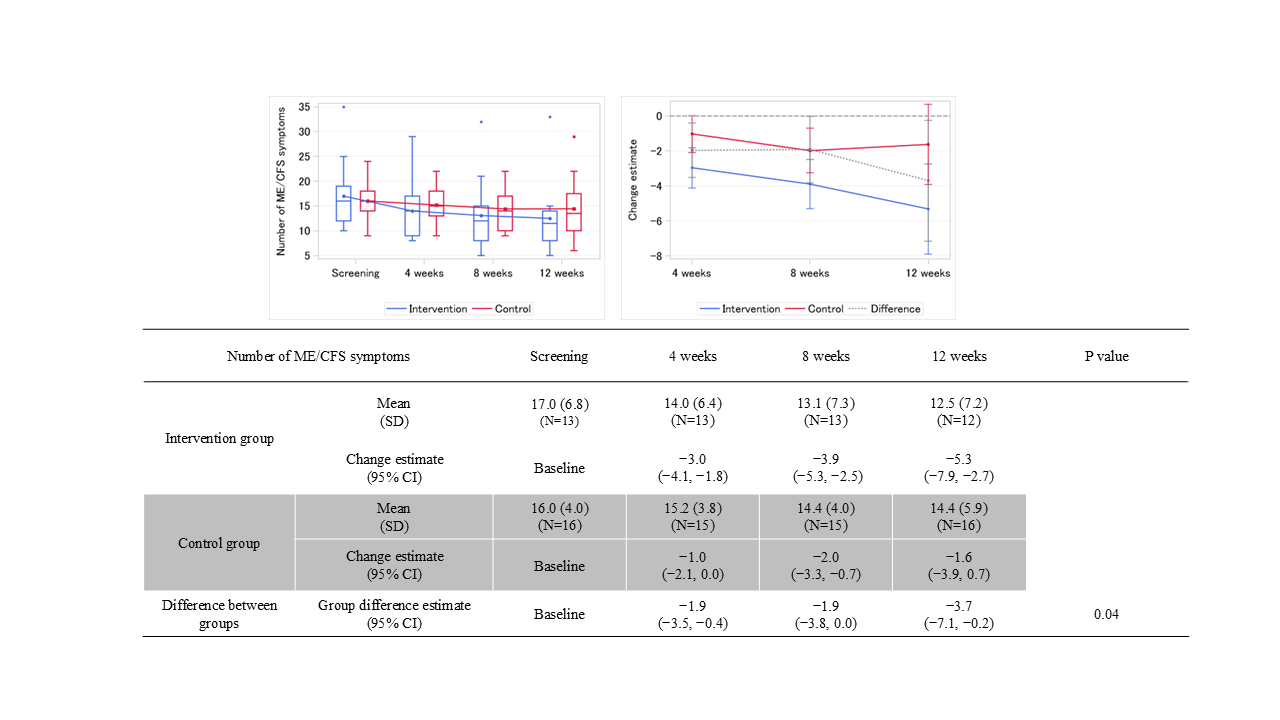

Supplement: Supplementary file 1 [file nutrients-18-00521-s001.zip › SupplementaryFigureS4.PNG]
